# Effective data-driven collective variables for free energy calculations from metadynamics of paths

**DOI:** 10.1093/pnasnexus/pgae159

**Published:** 2024-04-12

**Authors:** Lukas Müllender, Andrea Rizzi, Michele Parrinello, Paolo Carloni, Davide Mandelli

**Affiliations:** Department of Applied Physics, Science for Life Laboratory, KTH Royal Institute of Technology, SE-171 21 Solna, Sweden; Computational Biomedicine, Institute of Advanced Simulations IAS-5/Institute for Neuroscience and Medicine INM-9, Forschungszentrum Jülich GmbH, 52428 Jülich, Germany; Department of Physics, RWTH Aachen University, 52062 Aachen, Germany; Computational Biomedicine, Institute of Advanced Simulations IAS-5/Institute for Neuroscience and Medicine INM-9, Forschungszentrum Jülich GmbH, 52428 Jülich, Germany; Atomistic Simulations, Italian Institute of Technology, 16163 Genova, Italy; Atomistic Simulations, Italian Institute of Technology, 16163 Genova, Italy; Computational Biomedicine, Institute of Advanced Simulations IAS-5/Institute for Neuroscience and Medicine INM-9, Forschungszentrum Jülich GmbH, 52428 Jülich, Germany; Department of Physics, RWTH Aachen University, 52062 Aachen, Germany; Universitätsklinikum, RWTH Aachen University, 52062 Aachen, Germany; Computational Biomedicine, Institute of Advanced Simulations IAS-5/Institute for Neuroscience and Medicine INM-9, Forschungszentrum Jülich GmbH, 52428 Jülich, Germany

**Keywords:** enhanced sampling, collective variables, machine learning, path sampling, molecular dynamics

## Abstract

A variety of enhanced sampling (ES) methods predict multidimensional free energy landscapes associated with biological and other molecular processes as a function of a few selected collective variables (CVs). The accuracy of these methods is crucially dependent on the ability of the chosen CVs to capture the relevant slow degrees of freedom of the system. For complex processes, finding such CVs is the real challenge. Machine learning (ML) CVs offer, in principle, a solution to handle this problem. However, these methods rely on the availability of high-quality datasets—ideally incorporating information about physical pathways and transition states—which are difficult to access, therefore greatly limiting their domain of application. Here, we demonstrate how these datasets can be generated by means of ES simulations in trajectory space via the metadynamics of paths algorithm. The approach is expected to provide a general and efficient way to generate efficient ML-based CVs for the fast prediction of free energy landscapes in ES simulations. We demonstrate our approach with two numerical examples, a 2D model potential and the isomerization of alanine dipeptide, using deep targeted discriminant analysis as our ML-based CV of choice.

**Significance Statement**

The free energy landscape of complex (bio-)molecular processes can be described in enhanced sampling (ES) simulations as a function of suitable low-dimensional collective variables (CVs) which measure the progress of the process. Identifying the CVs can be very challenging, and, most often, this is the main bottleneck of these calculations. Here, by combining machine learning and enhanced path sampling, we can straightforwardly collect data on the physical pathways of the investigated processes in a robust manner. These data, which would be difficult to obtain with standard simulation approaches in conformational space, allow us to train highly efficient CVs, extending dramatically the domain of applicability of ES techniques for the investigation of biological and other complex systems.

## Introduction

Enhanced sampling (ES) methods (1, 2) are a powerful tool to investigate rare events in molecular systems, such as conformational changes of large biomolecular complexes, drug binding to receptor targets or phase transitions in materials (3–5). To obtain the free energy landscape describing these complex phenomena, a large class of ES methods work under the assumption that a few collective variables (CVs), functions s(R) of the atomic coordinates, exist that are able to provide a concise description of the transformation of interest. An external potential V(s) can then be defined, able to drive the rare transitions and allowing a reconstruction of the free energy profile. These methods include, among many others, umbrella sampling (6), hyperdynamics (7), well-tempered metadynamics and the On-the-fly Probability Enhanced Sampling (OPES) method (8–10), adaptive biasing force (11), or variationally enhanced sampling (12). We note here, that in cases where sufficiently long unbiased trajectories are available, researchers have designed general and elegant methods to describe the thermodynamics of a system without making use of CVs (13, 14). However, being able to access the Boltzmann distribution from unbiased molecular dynamics (MD) simulations is the exception rather than the rule.

The success of CV-based ES algorithms relies on the highly nontrivial choice of the CVs, which must be able not only to discriminate between the different metastable states but also, and most importantly, to describe the progress of the reaction. Recently, machine learning (ML)-based methods have been shown to be effective in delivering CVs that fulfill these two criteria (15–24). However, as in any ML approach, the results depend dramatically on the quality of the underlying data. This leads to a chicken-and-egg problem: for good results, one ideally needs data on the relevant metastable states and transitions between them, which, in turn, would require knowledge of a CV that allows their thorough sampling (25). As a result, most data-driven CV approaches still struggle to adequately accelerate the important motions in complex systems. To improve their efficiency, it has been previously recognized that including data from the transition state plays an important role in promoting the adequate description of the transition dynamics (26).

To harvest this essential data from the transition path ensemble, we shift our attention from enhanced sampling methods in configuration space to approaches focused on the direct sampling of the transition pathways. Among many such methods based on the statistical mechanics of trajectories (27–29), we consider the recently developed metadynamics of paths (MoP) algorithm (30). In contrast to the popular transition path sampling (31, 32), which has also been used for the identification of CVs (23, 24, 33, 34), MoP allows for the unconstrained exploration of multiple reactive paths connecting metastable states without the need for an initial path guess. This is achieved by performing metadynamics simulations in the space of all trajectories, making use of special CVs defined in *trajectory space* (CV*_t_* hereafter). As we will see below, a crucial property of MoP is its robustness in sampling the transition path ensemble with respect to a suboptimal choice of this CV*_t_*, considerably mitigating the chicken-and-egg problem described above.

**Fig. 1.**
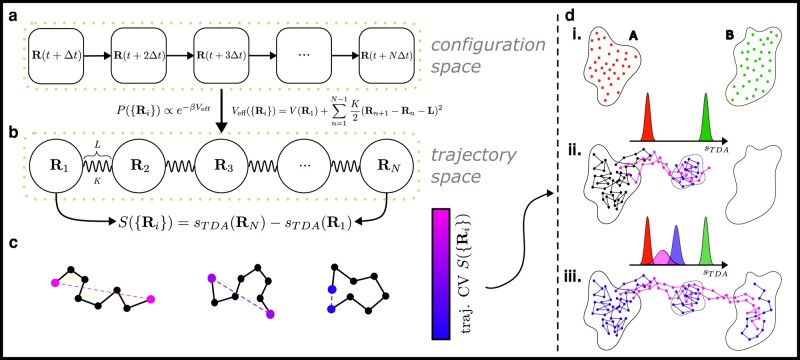
a) A discrete stochastic trajectory consists of the time series $\left\{R_{n}\right\}$ of configurations visited sequentially by the system. b) To each discretized trajectory can be assigned a well-defined Boltzmann-like statistical weight $e^{-\beta V_{\mathrm{eff}}(\{R_{n}\})}$. The effective potential is isomorphic to the potential energy of an elastic polymer. c) One can generate all possible discretized trajectories of a physical system by evolving the dynamics of a fictitious polymer subject to the force $F_{n}=-\nabla_{R_{n}}V_{\mathrm{eff}}$. Metadynamics can be used to accelerate the sampling of reactive pathways by choosing an appropriate CV defined in trajectory space. Here, we adopt as CV*_t_* the generalized polymer end-to-end distance *S* of Eq. 3, defined as the difference between the Deep-TDA CV*_c_* evaluated in the final and in the starting configuration of the polymer. d) Iterative procedure for DeepTDA CV*_c_* training. (i) Unbiased training datasets are generated in the initial (A) and final (B) states. A DeepTDA CV*_c_*  $s_{\mathrm{TDA}}$ is trained to map the two datasets into well-separated Gaussian distributions. (ii) A MoP run is performed and the generated paths are classified as trapped (blue and black) and reactive (violet) paths, based on the value of |S|. New datasets are identified from newly discovered metastable states and from configurations occupying the transition state region. A multistate DeepTDA CV*_c_* is trained to map these configurations into well-separated distributions and an updated end-to-end distance CV*_t_* is built. (iii) The desired outcome of a MoP run using the refined end-to-end CV*_t_* is depicted, showing sampling of trapped paths in all metastable states (in blue) as well as reactive pathways connecting different basins (in violet). This data can be used to design an optimal CV*_c_* in configuration space to converge the free energy landscape in a standard metadynamics simulation.

We show that the data obtained from MoP can be used to train an efficient ML-based CV in configuration space (CV*_c_* hereafter) to speed up standard metadynamics simulations. Here, among many different, powerful ML approaches, we use the deep targeted discriminant analysis (DeepTDA) supervised learning approach^a^ (35) previously developed to build CV*_c_*s. Briefly, DeepTDA trains a classifier that discriminates configurations belonging to different metastable states by mapping them into well-separated, user-defined locations in latent space. This approach can be used to incorporate not only data from multiple metastable states, but also from reactive trajectories connecting them (26, 35). The mapping is done such that the resulting 1D CV*_c_* describes the system’s progress from one basin to the other through the transition state region (see Fig. 1b and Materials and Methods for more details).

In practice, our proposed, iterative protocol (Fig. 1b) consists of the following steps:

**Step 1:** Standard MD simulations lead to kinetically trapped conformations in the (assumed to be known) initial and final basins (Fig. 1b). A CV*_c_* is obtained by training a DeepTDA model to discriminate these 2 states
**Step 2:** Starting from such CV*_c_*, a CV*_t_* is built and MoP simulations are performed
**Step 3:** The resulting trajectories are analyzed to identify newly discovered metastable states and reactive paths, and a new DeepTDA CV is trained including these data. If the latest MoP simulation found a path between initial and final states, the algorithm ends: it provides a complete map of the intermediate states and pathways of the molecular transform, which generally allows building efficient CV*_c_*. Otherwise, step 2 is repeated with the new CV*_c_*.

The method is designed to iteratively refine both CV*_c_* and CV*_t_*. More details on how the latter are constructed are provided below.

The article is organized as follows: after an introduction to MoP and the definition of CV*_t_*, we apply our iterative protocol (i) to a 2D model potential, used to test its applicability to multistate systems, and (ii) to the isomerization of alanine dipeptide in vacuum, which, despite its simpler two-state nature, provides a nontrivial test case on a molecular system.

##  

### Metadynamics of paths

In standard MD simulations, a discrete trajectory—consisting of the time series $\{R_{n}{\}}_{n=1,N}$ of configurations visited by the system—is generated in a *sequential* manner, due to the inherent seriality of the time evolution process (see Fig. 1a). MoP circumvents this problem—which rests at the base of the poor scaling of MD algorithms—and achieves parallelization in time by sampling directly from the phase space of all possible trajectories. The method applies to stochastic (Brownian) trajectories and exploits the isomorphism between the path probability distribution, p[A(R(t))], and the Boltzmann distribution of a fictitious elastic polymer (see Fig. 1b):


(1) $$p[A(R(t))]=\text{exp}\left[-\beta V_{\mathrm{eff}}(\{R_{n}\})\right]$$

(2) $$V_{\mathrm{eff}}=U(R_{1})+\sum_{n=1}^{N-1}\frac{K}{2}{\left(R_{n+1}-R_{n}-L_{n}\right)}^{2}.$$

In this equation, A is the Onsager–Machlup action (27), which is a functional of the (discretized) Brownian trajectory $R(t)=R_{1}\to R_{2}\to \cdots \to R_{N}$. $\beta =1/k_{B}T$, while K=mν/2Δt and $L_{n}=(\Delta t/m\nu )F_{n}$ are the effective spring constant and equilibrium length that depend on the physical parameters of the underlying Brownian dynamics: temperature *T*, mass *m*, damping coefficient *ν*, time step Δt (see Materials and Methods for details). *U* is the potential energy of the system and $F_{n}=-\nabla U(R_{n})$ is the physical force acting on the *n*th configuration.

Finite temperature MD simulations of the polymer are performed by computing the fictitious forces $F_{n}=-\nabla_{R_{n}}V_{\mathrm{eff}}$ acting on each configuration and are used to generate discretized trajectories distributed according to p[A(R(t))]. Metadynamics, in turn, can be used to focus the sampling on the important reactive trajectories connecting metastable states. This requires defining CV*_t_* in trajectory space.

Following Ref. (30), we define our CV*_t_* as the generalized end-to-end distance


(3) $$S(\{R_{n}\})=s(R_{N})-s(R_{1}),$$

where, in this work, s(R) is a DeepTDA CV*_c_*. The rationale for this specific choice of *S* is that it allows discriminating between elongated polymers (large values of |S|), which are likely to represent *reactive* trajectories, from kinetically *trapped* ones (with a low value of |S|), thus aiding in the discovery of new metastable states.

We note that reactive trajectories obtained from this method tend to spend more time in proximity to the transition states. This happens because the equilibrium spring constants are proportional to the physical force vector ($L_{n}\propto F_{n}$) and, therefore, tend to zero close to the stationary points of the potential energy surface, including the unstable saddle points (29). This feature increases the amount of data generated on transition states that can be used to train an efficient ML CV*_c_*.

### 2D model potential

**Fig. 2.**
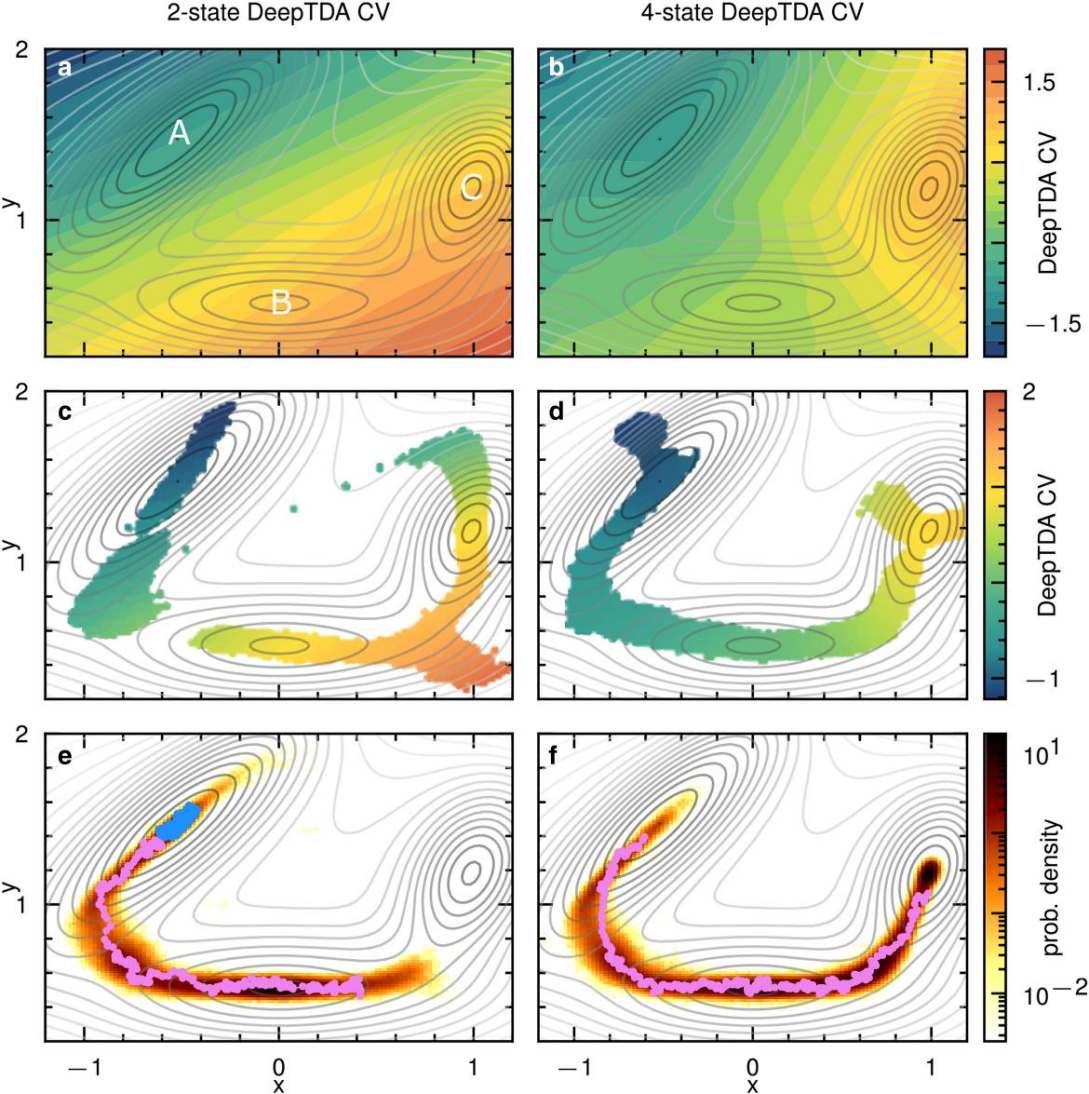
a) Colored map showing the values of the initial 2-state DeepTDA CV*_c_*. Initial, intermediate and final states are labeled as A, B, and C, respectively. b) Colored map of the 4-states DeepTDA CV*_c_*. Scatter plots of x,y coordinates are shown as obtained from an OPES simulation using c) 2-state and d) 4-state DeepTDA CV*_c_*. Points colored according to the corresponding CV*_c_* value. Configurations obtained from MoP simulations using e) the initial 2-state and f) the 4-state DeepTDA-based end-to-end distance CV*_t_* are shown. The violet and blue paths show the most probable reactive and trapped trajectories, respectively, i.e. the ones attaining the lowest value of the Onsager–Machlup action. In all panels, the isolines of the model potential are shown in gray as a reference.

We first applied our iterative protocol to a particle moving in the 2D model potential (adapted from Müller and Brown (36)) shown by the isolines of Fig. 2. The potential has three metastable states: an initial basin A and a final basin C which we assume to be known beforehand, and an intermediate basin B. The relative positions of the three minima were designed to provide a scenario in which neither coordinate axis can resolve the transition states and drive the exploration of the whole free energy surface. Furthermore, in this case, a neural network CV simply trained to discriminate between the A and C is likely to fail, as demonstrated below.

**Fig. 3.**
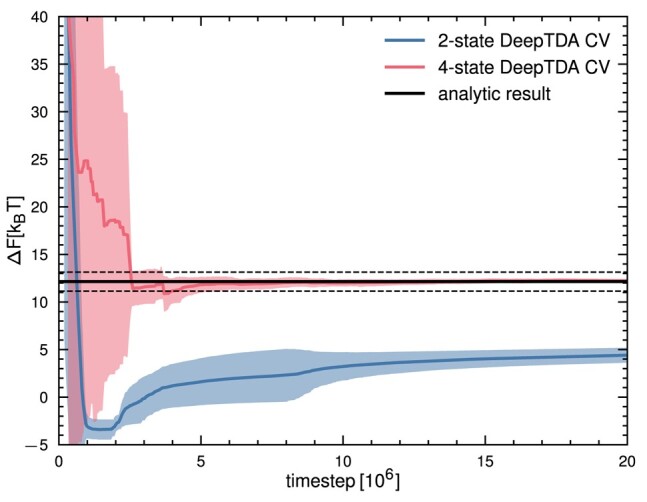
Free energy difference between the left and right basins of the model potential, as estimated from 5 independent OPES simulations biasing the 4-state DeepTDA CV, as a function of simulation time. The analytical result of $12.15\;k_{B}T$ was obtained by numerical integration over the MB potential (37) and is indicated in black, with dotted lines indicating a margin of $1\;k_{B}T$.

We first performed unbiased simulations in the A and C basins and trained an initial 2-state DeepTDA CV*_c_*. The value of the latter is shown by the colored map reported in Fig. 2a. Clearly, the intermediate metastable state B is not discriminated from the C basin since the CV attains the same value in the two basins. As a consequence, when used in an OPES simulation, we found that this CV*_c_* is very inefficient in guiding transitions between A and C. Furthermore, during the simulation, the system is driven to sample unphysical trajectories that differ greatly from the minimum energy pathway (see Fig. 2c). As a result, we also observe that the free energy difference between basins A and C is not accurately estimated when compared to the analytical result obtained by numerical integration of the potential (see Fig. 3).

Nevertheless, we can employ this suboptimal CV to construct the end-to-end distance CV*_t_* defined in Eq. 3 for use in a MoP simulation. The samples obtained from the simulation in trajectory space are reported in Fig. 2e. Notably, the sampled trajectories follow the underlying minimum energy pathways. This is due to the forces driving the polymer dynamics not being directly related to the potential energy surface but rather to the Onsager–Machlup action, which is lower for the more statistically relevant ones. By using the end-to-end distance CV*_t_*, the metadynamics bias acts only on the polymer endpoints, while the intermediate replicas are free to relax, minimizing the OM action. This illustrates the robustness of MoP in sampling physically relevant trajectories even when using suboptimal CV*_t_*. Importantly, the analysis of the data allows blindly detecting the intermediate state from the presence of crumpled polymers confined entirely into this basin, which are characterized by small values of the end-to-end distance CV*_t_*, S∼0.

Partial reactive trajectories connecting the A and B basins were also observed (see Fig. S4b). However, the simulation could not sample complete reactive paths connecting from A to C due to the suboptimal CV*_c_* used in Eq. 3, which cannot distinguish correctly between B and C. We solve this problem by performing a second iteration of the algorithm in which the information gained from the MoP run is used to train a refined, 4-states DeepTDA CV, including data from the three metastable states plus the transition region between A and B (for all technical details we refer to the Materials and Methods). The colored map of the new CV*_c_* is shown in Fig. 2b. It is apparent that all relevant metastable and transition states are resolved.

The new CV*_c,t_* drives complete transitions from A to C both when used in MoP (Fig. 2f) as well as in standard OPES simulations (Fig. 2d). Figure 3 shows that the free energy difference between basins A and C, as estimated with the new CV*_c_*, is in excellent agreement with the analytical result. We also checked that the corresponding end-to-end distance CV*_t_* improves sampling in trajectory space. Figure 2f reports the result of a MoP simulation, showing the sampling of complete reactive trajectories connecting A and C along the minimum free energy path. The efficiency of this CV is further demonstrated by the fact that it was able to generate also the partial paths connecting basins A and B, and B and C (see Fig. S5b). From the complete reactive paths, we can also observe that they indeed spend an increased amount of time in the vicinity of the transition state, as illustrated in Fig. S6.

The new dataset obtained from MoP allowed us to train a 5-states DeepTDA CV*_c,t_*, also including data from the transition state between B and C. The resulting CVs, however, did not lead to significant improvements respect to the 4-states versions.

### Alanine dipeptide

**Fig. 4.**
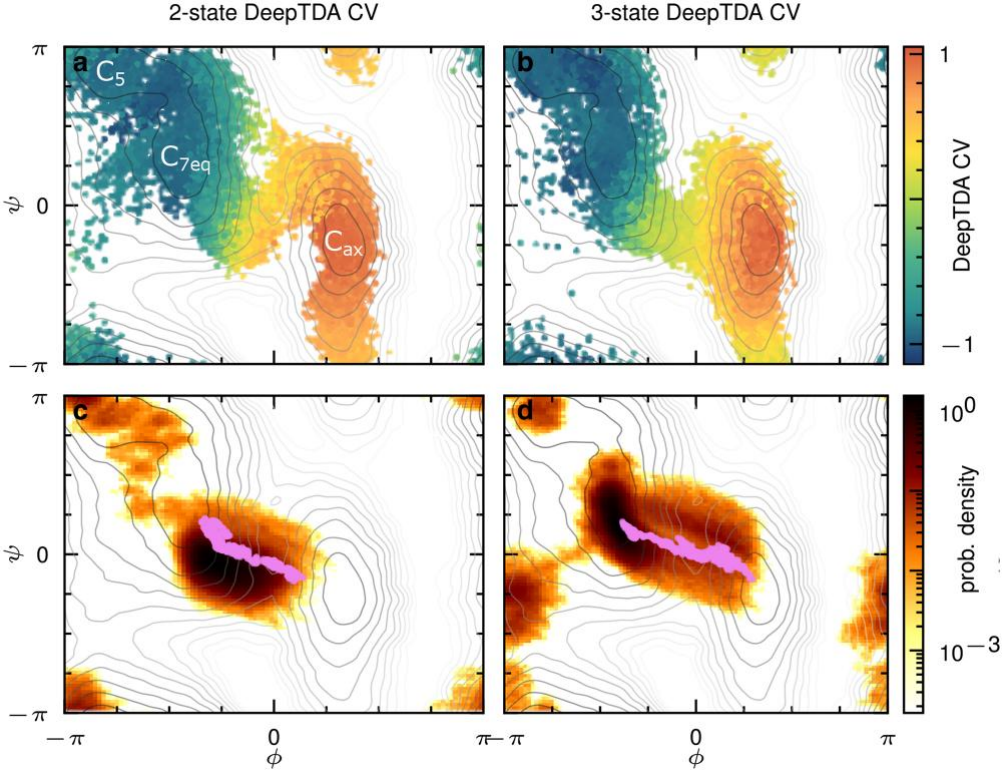
Biased simulations in configuration and trajectory space of alanine dipeptide. Scatter plots of x,y coordinates in OPES simulation using a) 2-state and b) 3-state DeepTDA CV*_c_* are shown, colored according to CV value. MoP simulations using c) initial and d) 3-state DeepTDA CV*_t_* are shown, shown as normalized density of all configurations in logarithmic scale. The violet paths show the most probable reactive trajectories, i.e. the ones attaining the lowest value of the Onsager–Machlup action. In all panels, isolines of the FES obtained from a reference calculation are shown in gray.

We now move to the conformational dynamics of alanine dipeptide in vacuum. The free energy surface in the Ramachandran plane spanned by the dihedral angles *ϕ* and *ψ* is indicated by the gray isolines in Fig. 4. The system is characterized by the presence of three metastable states, labeled $C_{5}$, $C_{7eq}$, and $C_{ax}$. Specifically, the $C_{5}$ and $C_{7eq}$ conformers are separated by a barrier of the order of a few $k_{B}T$ (38) and form a unique basin at room temperature, while a minimum barrier of around 13 $k_{B}T$ separates $C_{7eq}$ and $C_{ax}$.

**Fig. 5.**
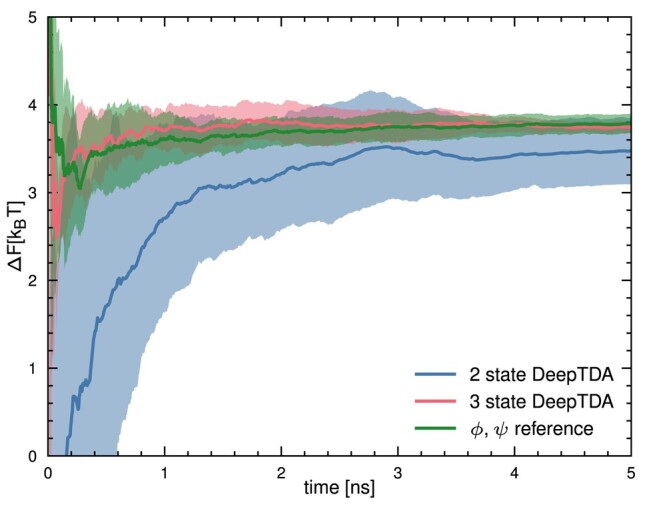
Free energy difference between the $C_{7eq}$ and $C_{ax}$ basins of alanine dipeptide over time. Shown are the results obtained from 5 independent enhanced sampling simulations respectively, biasing the 2-state and 3-state DeepTDA CV, as well as reference calculation using the dihedral angles ϕ,ψ as CV.

As done in the previous example, we start by performing unbiased simulations in the two metastable states, and training an initial 2-state DeepTDA CV*_c_*. In agreement with Ref. (35), we found that this CV is already able to drive transitions across the barrier separating $C_{7eq}$ and $C_{ax}$ to an acceptable degree when used in an OPES simulation (see Fig. 4a). However, in doing so, the system does not follow precisely the expected minimum free energy path but it samples also trajectories crossing high energy barriers around ψ∼π/2. This can be explained by the lack of transition state data (35). Figure 5 reports a comparison of the performance of this CV*_c_* with the results obtained from a reference OPES simulation performed biasing the *ϕ* and *ψ* angles, showing slow convergence and a significant discrepancy of $0.2k_{B}T$ in the free energy difference after 5 ns of simulation time.

Figure 4c shows the result of MoP using the corresponding CV*_t_* in trajectory space, built following Eq. 3. It can be seen that the sampling is focused on trajectories which follow the minimum free energy path. However, diffusion between the basins $C_{5}$ and $C_{7eq}$ is slow and only one transition takes place. This is due to the fact that these two minima are not distinguished by the DeepTDA CV*_c_*. Furthermore, no paths reach fully into the $C_{ax}$ basin. Again, this can be explained by the lack of information on the transition state, which causes the starting CV to reach its maximum value before the true potential minimum is reached. Nonetheless, we can extract data from the transition state from the sampled reactive paths to train a new, 3-state DeepTDA CV*_c_* (see Materials and Methods for details). In Fig.4b, we observe that in configuration space, transitions are now confined more closely to the minimum free energy paths, improving the efficiency of the CV*_c_*. This is further supported by Fig. 5, demonstrating improved convergence speed and smaller statistical fluctuations of the 3-state DeepTDA CV*_c_*, as compared to the 2-state DeepTDA CV*_c_*, and similar convergence speed as the reference calculation. When used in a MoP simulation, reactive paths are sampled that reach considerably further inside the $C_{ax}$ state (see Fig. 4d) and fully connecting the $C_{7eq}$ and $C_{ax}$ basins. We also note that, after starting in the $C_{5}$ basin, the simulation takes the less likely, but known path (29) across the higher barrier between $C_{5}$ and $C_{7eq}$, before sampling $C_{7eq}$ and reactive paths between $C_{7eq}$ and $C_{ax}$. However, these configurations are sampled during the initial part of the OPES-based MoP simulation—where the bias deposition is particularly aggressive—and are therefore exempted from further analysis.

### Conclusions

We have presented an iterative approach based on the metadynamics of paths algorithm (30) to reconstruct free energy landscapes as a function of data-driven CVs using datasets supplemented with configurations from the transition state ensemble. In doing so, we have also addressed directly the problem of designing efficient CVs in trajectory space. We found that the augmentation with MoP data leads to a significant performance increase of the learned CVs in configurational space. Good CVs could be generated even when using MoP in an exploratory manner (i.e. convergence was not needed), thus considerably reducing the computational effort required to obtain meaningful results.

Besides being the only path sampling method so far enabling the exploration of free energy landscapes using CVs, the use of MoP to obtain transition state data offers several other advantages: (i) in contrast to previous approaches (23, 24, 26, 34), MoP enables the sampling of (non)reactive trajectories in an unconstrained manner and is robust to the choice of sub-optimal CVs; (ii) running MoP amounts to a single metadynamics simulation, considerably simplifying the methodology compared to Monte Carlo approaches like transition path sampling; (iii) MoP can be implemented in an extremely parallel fashion (39) to exploit modern massively parallel supercomputers (which have recently breached the exascale limit); (iv) compared to chain-of-states methods (29, 40, 41) MoP generates a large amount of configurations, which is required for data-driven applications like ours.

The efficiency of our procedure was tested in two models of increasing complexity, including a simple molecular system. We expect our protocol to aid in the discovery of CVs, the exploration of transition pathways, and the estimation of free energy profiles in complex systems of relevance in biology, chemistry, and materials science. Future work will focus on scaling the proposed protocol to more challenging, real-world applications. While MoP can be readily applied to the condensed phase, including explicit solvent, applications to large macromolecules of our ML approach will need a suitable set of input descriptors such as backbone dihedral angles, per-residue Q-values, or water coordination numbers (42, 43).

Finally, we note that, in this work, we have focused on building ML CVs that use only the spatial distribution of the samples obtained from MoP data. However, learning dynamical information from the trajectory data (18, 19, 44) is another very appealing avenue, as the sampled paths carry information on the unbiased dynamics of the system.

## Materials and methods

### DeepTDA CVs

The DeepTDA neural network CVs (35) are trained in PyTorch using an existing implementation in the mlcolvar package (45), in its release v0.2.0. The networks use a feed-forward architecture with 3 hidden layers with {24,12,1} nodes and the ReLU activation function. To enforce the target distribution on the latent CV space, the objective function for the neural network is chosen to be a mean-squared error between the mean and standard deviation at the hidden layer and their respective target values, $\mu^{tg}$ and $\sigma^{tg}$, which in the 1D case is given by,


(4) $$L=\sum_{k}^{N_{s}}\alpha (\mu_{k}-\mu_{k}^{tg}{)}^{2}+\beta (\sigma_{k}-\sigma_{k}^{tg}{)}^{2},$$

where *k* denotes the $N_{s}$ different states, and the hyperparameters *α* and *β* ensuring adequate scaling of the respective loss terms. The resulting DeepTDA CV is normalized over the training data to a range of −1≤s≤1.

In all trained networks, the parameters are optimized using the ADAM optimizer (46) with a learning rate of $10^{-3}$. In addition to the TDA loss in Eq. 4, L2 regularization with $\lambda =10^{-5}$ has been added to the weights. The training is stopped when convergence of the loss function is reached, using an Early-Stopping routine with patience set to 15 epochs to avoid overfitting. The hyperparameters *α* and *β* were set to values of 1 and 250, respectively.

### 2D model potential

To evaluate the proposed methodology, we designed a 2D model potential, based on the analytical form introduced by Müller and Brown (36). The isolines of this modified potential are shown in Fig. S1 and has the analytical from


(5) $$\begin{array}{rl} U_{\mathrm{MB}}(x,y) & =\sum_{k}A_{k}\text{exp}[a_{k}(x-x_{k}^{0}{)}^{2}\\ & \;+b_{k}(x-x_{k}^{0})(y-y_{k}^{0})+c_{k}(y-y_{k}^{0}{)}^{2}],\\ A & =(-16,-11,-17,2),\\ a & =(-10,-1,-6.5,0.4),\\ b & =(5,0,11,0),\\ c & =(-5,-10,-6.5,1.1),\\ x_{0} & =(1,0,-0.5,0),\\ y_{0} & =(1.2,0.5,1.5,1). \end{array}$$

All simulations in this work are started in basin A, where the potential assumes its global minimum. In this work, we consider Langevin dynamics of a single particle moving along the model potential. The simulations use natural units, such that $k_{B}=1$, and a temperature of T=0.1, placing the highest free energy barrier at around $120\;k_{B}T$.

Because the analytical form of the potential is known, the difference in free energy between the basins A and C can be calculated directly via numerical integration, with


(6) $$\Delta F=-\frac{1}{\beta }\log\;\frac{\int_{C}e^{-\beta U_{\mathrm{MB}}(x,y)}\;dx\;dy}{\int_{A}e^{-\beta U_{\mathrm{MB}}(x,y)}\;dx\;dy},$$

where A={(x,y)∣y>x+1.5} and C={(x,y)∣x−1.5<y<x+1.5}, which in this case equates to $12.15\;k_{B}T$.

The simulations in configurational space of the 2D model potential were performed using the molecular simulation engine LAMMPS (47) in its stable release version from 23. June 2022, patched with PLUMED 2.8 (48), which also provides an interface with the LibTorch C++ library to implement the neural network-based DeepTDA CVs (45). The damping constant in the Langevin thermostat is set to a value of 0.1, which corresponds to a value of ν=10, and the time step is set to Δt=0.01 in arbitrary units of time.

To perform enhanced sampling simulations, we here use the recently introduced OPES method (10), an implementation for which is provided in PLUMED. For detailed information about the usage of this bias potential and a definition of the relevant parameters, the reader is referred to the PLUMED documentation. For the OPES simulation with the initial 2-state DeepTDA CV, the parameters are set to BARRIER =17, PACE =300, and SIGMA =0.03. The SIGMA is chosen to reflect the standard deviation of the CV values in a short, unbiased simulation. For OPES simulation using the 4-state DeepTDA CV, the parameter values BARRIER =15, PACE =300, and SIGMA =0.01. The SIGMA parameter is again chosen as the CVs standard deviation in unbiased simulation, and the BARRIER parameter is lowered to allow for faster convergence.

To carry out the Metadynamics of Paths simulations in trajectory space, an extra fix is added to the LAMMPS suite, adapted from Ref. (30) to directly evaluate the analytical derivatives of the potential. The friction and time step parameter of the polymer are set to ν=10 and Δt=0.01, respectively. The polymer length should be chosen to allow sampling the reactive trajectory of interest. Here, a value of N= 288 was found to be sufficient. Note that thanks to its parallel implementation, the computational cost per time step of the MoP algorithm only weakly depends on the polymer size (see Fig. S2). In trajectory space, the polymers are then sampled at a time step of 0.01 and a damping constant of damp =100. The initial configuration for the polymer was obtained by running an unbiased MetaD of Paths simulation starting from the same configuration for every bead at $r=(-0.5,1.5{)}^{⊺}$ and letting the polymer relax for $10^{4}$ steps. While relaxing the polymer removes the dependence on the precise starting conformation, we found that it is necessary to run a sufficient number of relaxation steps in order to achieve a faster exploration of relevant reactive trajectories.

To enhance the sampling of the polymers, OPES is again used. The underlying configurational DeepTDA CV is first evaluated on each bead. Then, a modified version of the CUSTOM/MATHEVAL action is used to access the values from different beads, to evaluate the end-to-end difference for use as the trajectory CV. MoP simulations with both the initial DeepTDA CV and the 4-state CV are performed with OPES parameters set to BARRIER =20 and PACE =500, and an adaptive SIGMA. In both cases, the MoP simulations were run for $2\times 10^{6}$ steps, saving polymer configurations every 500 steps.

### DeepTDA CV training for 2D model potential

For the initial DeepTDA CV $s_{0}$ on the unbiased training data, the target centers and widths are chosen as $\mu^{tg}=[-7,7]$ and $\sigma^{tg}=[0.2,0.2]$, respectively. The training dataset consists of 6,000 configurations from each of the initial metastable states, as shown labeled “unbiased data” in Fig. S3b.

We now train the 4-state DeepTDA CV in a two-step process, the reason for which is explained below. From the trajectory data, shown in Fig. 2e, we first isolate the reactive trajectories and those trapped in metastable states. This can be quantified by using the trajectory CV $S(\{R_{i}\})$ in Eq. 3, and we here classify as kinetically trapped or confined trajectories with values of S≤0.3. In this way, we find trajectories trapped in the initial basin A, but also those confined to the previously “unknown” basin B. To separate these configurations between known and unknown states, OPTICS clustering (49) is used.

To filter the reactive trajectories, the range of values to select for depends strongly on the respective system under consideration, and how well the initial DeepTDA CV is able to discern different regions of the phase space, which is why we first train a 3-state DeepTDA CV $s_{1}$. For the training of this CV, target centers and widths for this CV are chosen as $\mu^{tg}=[-15,0,15]$ and $\sigma^{tg}=[0.3,1.0,0.3]$, respectively, corresponding to a respective separation of Δ=15. Evaluating this CV on the previously obtained trajectory data, we can now much more confidently set a threshold of $|S\{R_{i}\}|\geq 1$ to select trajectories that connect A and B. Now, information from the reactive paths is taken into account, by selecting configurations that satisfy $\mu_{A}+3\sigma_{A}\leq s_{1}(r)\leq \mu_{\mathrm{int}}-3\sigma_{\mathrm{int}}$, where the $\mu_{A/\mathrm{int}}$ and $\sigma_{A/\mathrm{int}}$ denote mean and standard deviation in the initial/intermediate metastable states, respectively. The precise multiple of the standard deviation should be chosen carefully, as to select data that covers as wide a CV*_c_* range as possible, without introducing overlaps. These data are added as a fourth state for the training of the 4-state DeepTDA CV $s_{2}$, using the target widths $\mu^{tg}=[-30,-15,0,15]$ and centers $\sigma^{tg}=[0.3,4.0,1.0,0.3]$, and the training data are shown in Fig. S3a. These values are chosen by visual inspection of the CV histograms in the training data, to select for sharp peaks in the metastable states and broader, slightly overlapping distributions for the reactive paths and transition states (see Fig. S3b). In both cases, a random subset of 6,000 configurations is selected from the trapped and reactive trajectory data, to match the amount of unbiased data. A comparison of the performance of the 2- and 4-state DeepTDA CVs in an OPES simulation in configuration space is shown in Fig. S7. In the supplementary material, we also provide snapshots of the polymer configurations from MoP simulations with both CVs (see Fig. S8) and a supporting movie M1 showing the evolution of the MoP simulation using the 4-state DeepTDA CV. We also show the distribution of |S| values for both CVs (see Fig. S9), as well as results of MoP simulations starting in the metastable basins B and C (see Fig. S10).

### Alanine dipeptide

For the simulations of the conformational dynamics of alanine dipeptide in vacuum, we again used LAMMPS patched with PLUMED. The Amber99-SB (50) force field was used, converted for use in LAMMPS using the convert.py script in the InterMol (51) software. We consider Langevin dynamics in an NVE ensemble at a temperature of 300 K, and use a time step of 0.5 fs and a dampening constant of 500 fs. To perform biased simulations with the DeepTDA CVs in OPES, we used parameter values BARRIER =50, PACE =100, and an adaptive SIGMA.

To carry out MoP simulations, we used the path_dynamics fix provided by and described in Ref. (30). The parameters of the polymer are set to $\nu =0.25\frac{1}{s}$ and Δt=1fs. In trajectory space, the polymers, made up of N=512 beads, are then sampled at a time step of 1.0 and a damping constant of damp =1,000 in trajectory space. The initial configuration for the polymer was obtained by running an unbiased simulation in configuration space for $10^{6}$ steps, and saving the last 512 steps as initial configurations for the polymer beads. The polymer was then allowed to equilibrate in an unbiased MoP simulation for $10^{6}$ steps. As described above, we again use OPES to drive the sampling of the polymers with a bias potential, using parameter values BARRIER =80 and PACE =1,000, as well as a bias factor of 15. We again ran MoP simulations for $4\times 10^{6}$ steps for both realizations of the DeepTDA CV, saving polymer configurations every 1,000 steps.

The DeepTDA neural network-CVs for alanine dipeptide are trained with largely identical settings to the case of the model potential, with the exception of a learning rate of $10^{-3}$. As a set of descriptors, we use the set of pairwise distances between the heavy atoms of alanine dipeptide, as compiled by Bonati et al. (17), thereby insuring rototranslational invariance of our CV.

The 2-state DeepTDA CV $s_{0}$ is trained with 4,000 configurations each from the $C_{5}/C_{7eq}$ and $C_{ax}$ basins, respectively. As values for the target centers and widths for the modes in CV spaces, we again choose $\mu^{tg}=[-7,7]$ and $\sigma^{tg}=[0.2,0.2]$. Proceeding as described in the main text, by first separating the trajectory data, sampled with MoP using the 2-state CV, into trapped paths with a value of $S(\{R_{i}\})\leq 0.5$ and reactive paths with $S(\{R_{i}\})\geq 1.2$ (see Fig. S12). In this case, we do not observe any confined data in previously unexplored regions, so we proceed by integrating data from the transition state as an intermediary. The training data for the 3-state DeepTDA CV $s_{1}$ is thus chosen by selecting configurations with $s_{0}\geq 0.3$ from the reactive paths, as shown in Fig. S11. From this data, we again choose a random subset of 4,000 configurations to match the amount of unbiased data, and train the new CV by choosing target centers and widths $\mu^{tg}=[-7,0,7]$ and $\sigma^{tg}=[0.3,1.0,0.3]$. Trapped and reactive trajectories identified from a MoP simulation using this new CV are reported in Fig. S13, and a comparison of the performance of the 2- and 3-state CVs in an OPES simulation in configuration space is given in Fig. S14. In the supplementary material, we also provide snapshots of the polymer configurations from MoP simulations with both CVs (see Fig. S15) and a supporting movie M2 showing the evolution of the MoP simulation using the 3-state DeepTDA CV. We also show the distribution of |S| values for both CVs (see Fig. S16), as well as results of a MoP simulation starting in the metastable state $C_{\mathrm{ax}}$ (see Fig. S17).

## Supplementary Material

pgae159_Supplementary_Data

## Data Availability

The input files for LAMMPS and PLUMED used to generate the presented results, scripts to compile the necessary software, as well as example code to train the DeepTDA NN CVs, can be found on GitHub at https://github.com/lmuellender/MoP-DeepTDA. Additionally, all data and files are available via Zenodo at https://doi.org/10.5281/zenodo.10845989.

## Note

a Note that the choice of DeepTDA was made here mainly because of its simplicity and straightforward interpretation. However, the data generated by MoP could be used to train many other data-driven methods (18–21, 23, 24).

## Preprints

This article was posted to a preprint: https://doi.org/10.48550/arXiv.2311.05571
