# Supplementary material for: Effective data-driven collective variables for free energy calculations from metadynamics of paths
Source: PNAS Nexus. 2024 Apr 12;3(4):pgae159. doi: 10.1093/pnasnexus/pgae159 (PMC11044970; doi:10.1093/pnasnexus/pgae159)
Supplement: pgae159_Supplementary_Data [file pgae159_supplementary_data.zip › supp_info_final.pdf]

Supporting Information for

Effective Data-Driven Collective Variables for Free Energy Calculcations from Metadynamics of Paths

Lukas Müllender, Andrea Rizzi, Michele Parrinello, Paolo Carloni, Davide Mandelli

Corresponding authors: Paolo Carloni and Davide Mandelli.
E-mail: p.carloni@fz-juelich.de or d.mandelli@fz-juelich.de

This PDF file includes:

Figs. S1 to S17
SI References

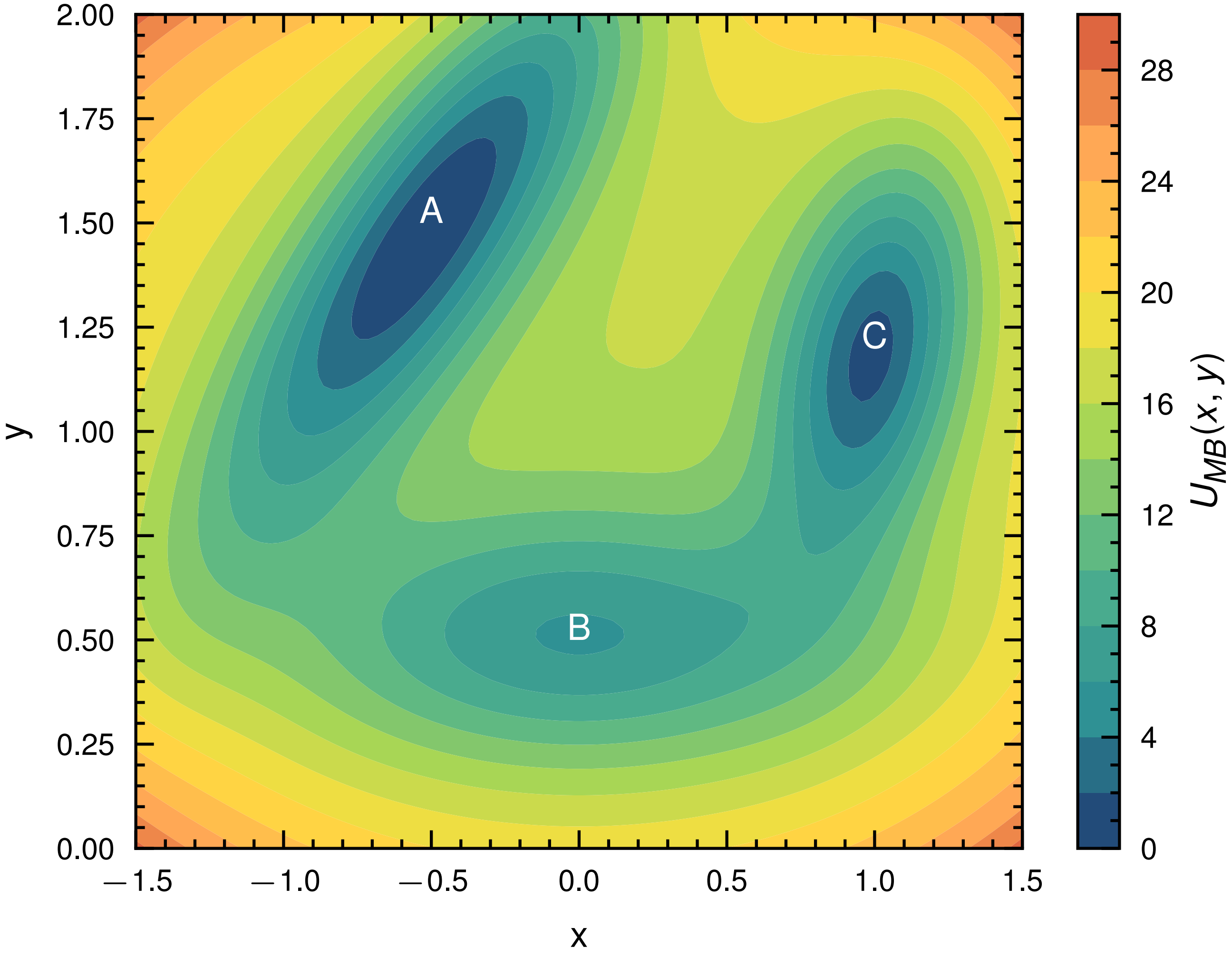

Fig. S1. Isolines of the 2D model potential energy surface.

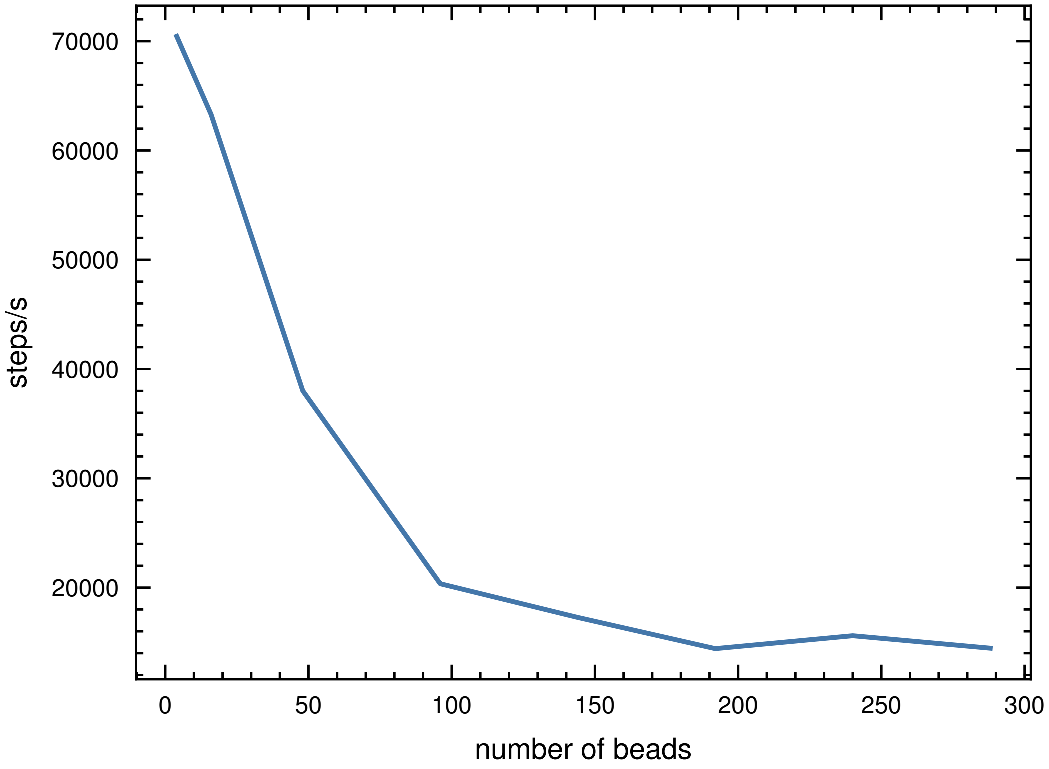

Fig. S2. Performance of MoP as a function of the number of beads in the polymer, measured in MoP steps per second, running with one bead per MPI task and one CPU core per bead. The performance decreases from 70k steps/s for a very small polymer made of 4 beads to 14k steps/s for a polymer made of 200 beads, after which a plateau is reached that extends up to the number of N=288 beads used in our simulations. Note that, while the performance decreases by a factor of 70/14=5, the number of beads in the polymer has grown by a factor of 288/4=72, which is more than one order of magnitude larger.

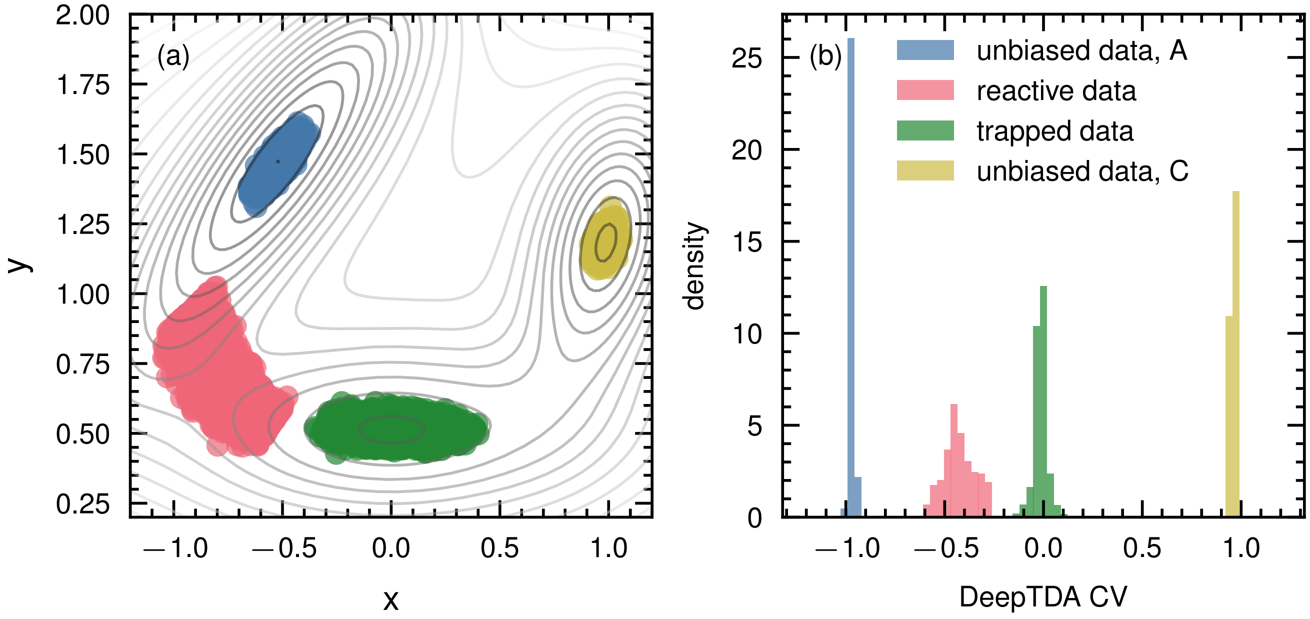

Fig. S3. Training data and histogram of CV distributions for training the 4-state DeepTDA CV in the case of the 2D model potential. (a) Scatter plot of the training data, with data from unbiased simulations in the left and right basins shown respectively in blue and yellow, selected configurations from reactive paths generated by MoP shown in pink and configurations from trapped paths in the intermediate basin shown in green. From all 4 states, 6666 configurations are shown and used to train the 4-state DeepTDA CV. Isolines of the 2D model potential are indicated in grey. (b) Histogram of the trained 4-state DeepTDA CV evaluated on the training data from the different basins. Note how the unbiased data are mapped to sharp peaks at the end points, and data from reactive and confined trajectories are mapped to broader distributions.

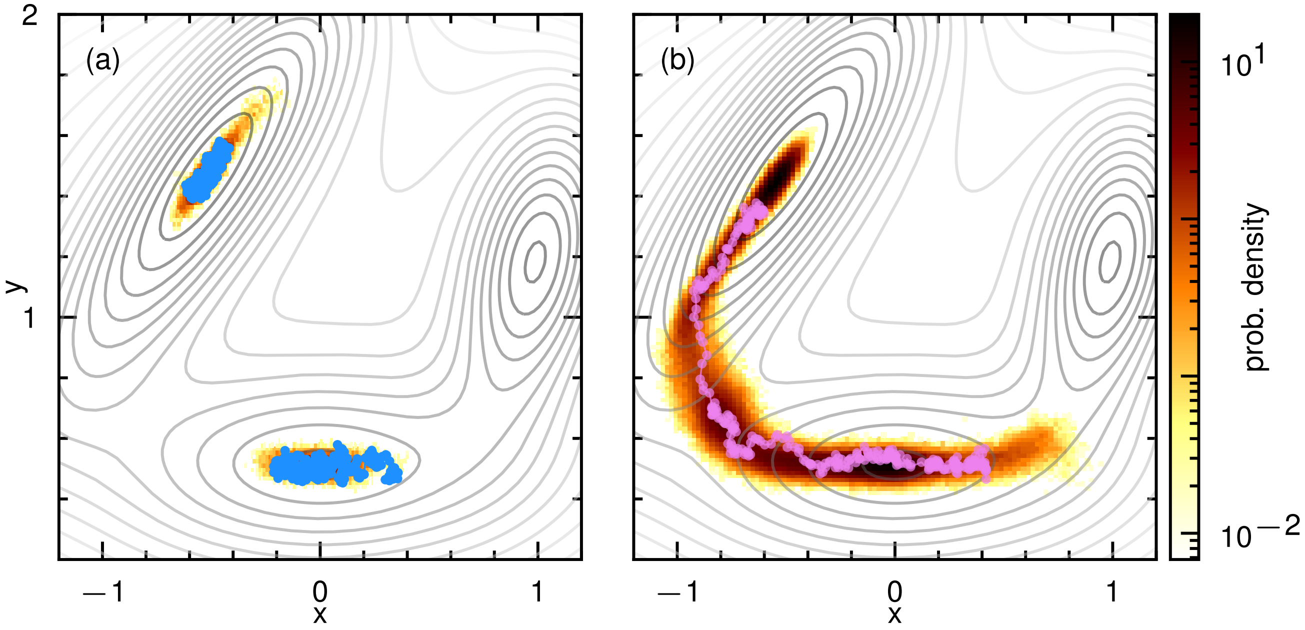

Fig. S4. Trapped (a) and reactive (b) trajectories, isolated from a MoP simulation using the 2-state DeepTDA CV for the 2D model potential in trajectory space. Examples of trapped trajectories are shown in blue, a reactive trajectory in pink. The densities are calculated from 152 trapped and 1855 reactive trajectories, respectively. Isolines of the potential are indicated in grey.

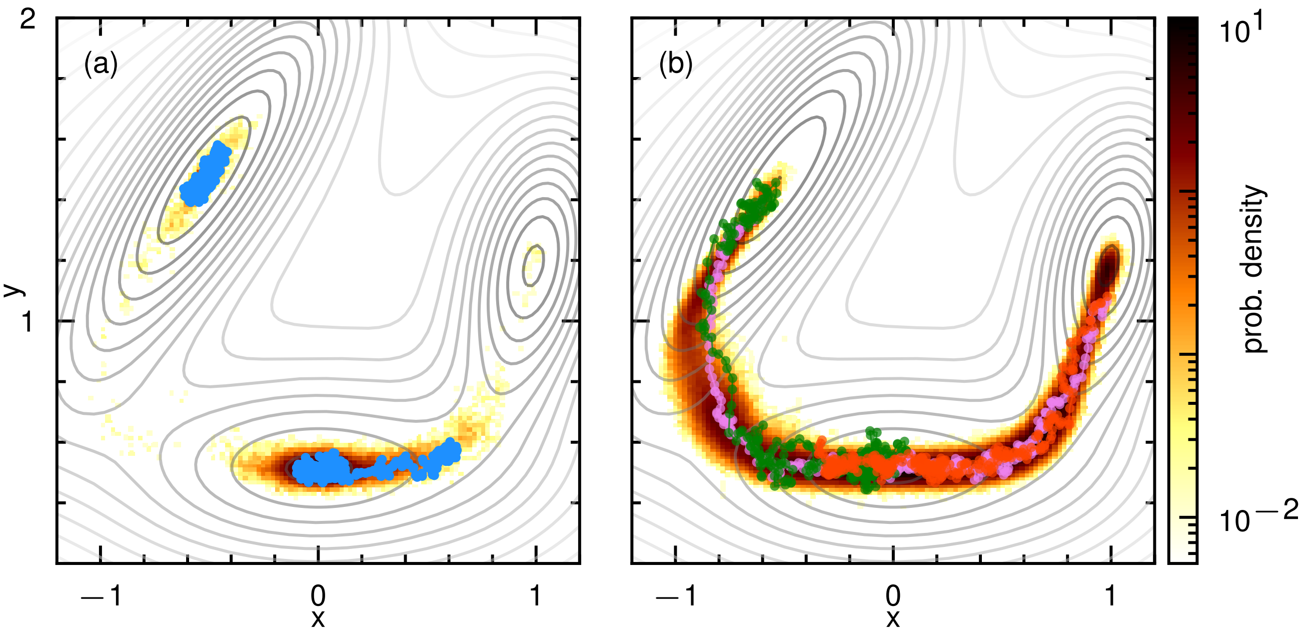

Fig. S5. Trapped (a) and reactive (b) trajectories, isolated from a MoP simulation using the 4-state DeepTDA CV for the 2D model potential in trajectory space. The densities are calculated from 208 trapped and 2603 reactive trajectories, respectively. Examples of trapped trajectories are shown in blue. Reactive trajectories connecting the basins A-B, A-C and B-C are shown in green, pink and red, respectively. Isolines of the potential are indicated in grey.

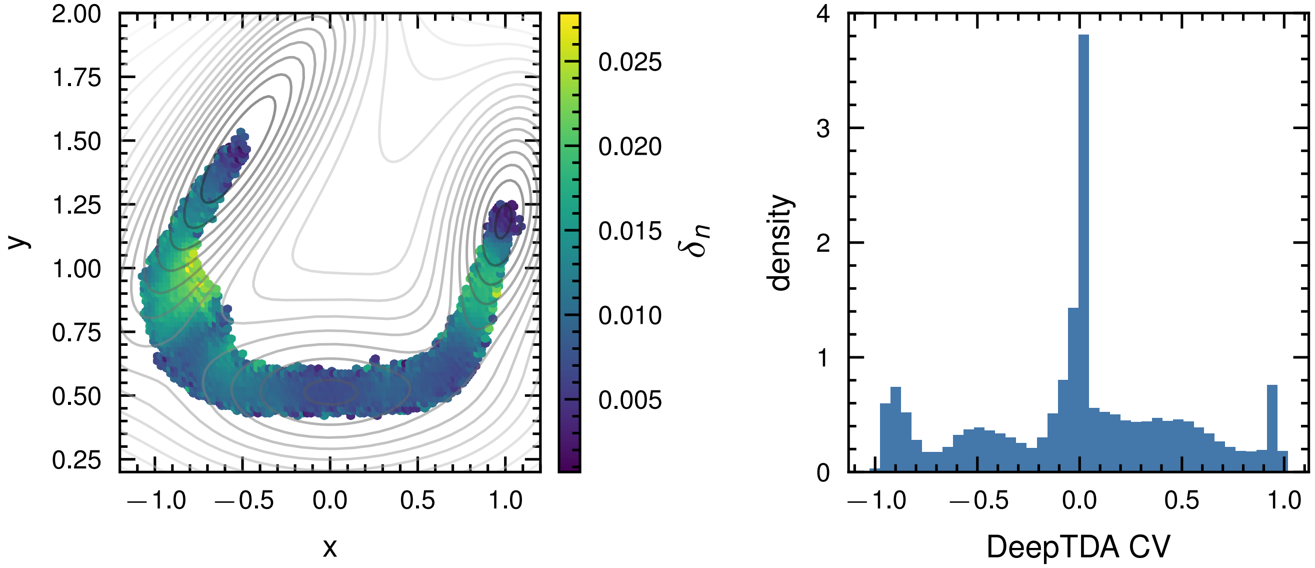

Fig. S6. Trajectories sampled by MoP spend more time in the vicinity of transition states. (a) Moving average of the oriented displacement between adjacent beads in a trajectory (average adjacent displacement, or AAD). If $\{\mathbf{d}_n\}_{n=1}^{N}$ is a set of descriptors or CV$_c$s, at bead n this measure is given by $\delta_n = \left| \frac{1}{2k} \sum_{m=-k}^{k} \mathbf{d}_{n-m+1} - \mathbf{d}_{n-m} \right|$, where k is the kernel width of the moving average. Thereby, it directly shows regions of *increased* density along the polymer as regions of *decreased* AAD. Here it is evaluated along the reactive trajectories obtained from a MoP simulation using the 4-state DeepTDA CV$_t$, as described in the main text, with $k = 15$. Note that AAD is decreased not only in the known metastable states A and C, but also in state B and on the transition states connecting them. (b) Histogram of the 4-state DeepTDA CV, evaluated on the reactive paths obtained in a MoP simulation. Note the peaks in the distribution around $s = -1, 1$ corresponding to basins A and C, the sharp peak at $s = 0$ corresponding to the intermediate basin B, and peaks around $s = -0.5, 0.5$, corresponding to the transition states.

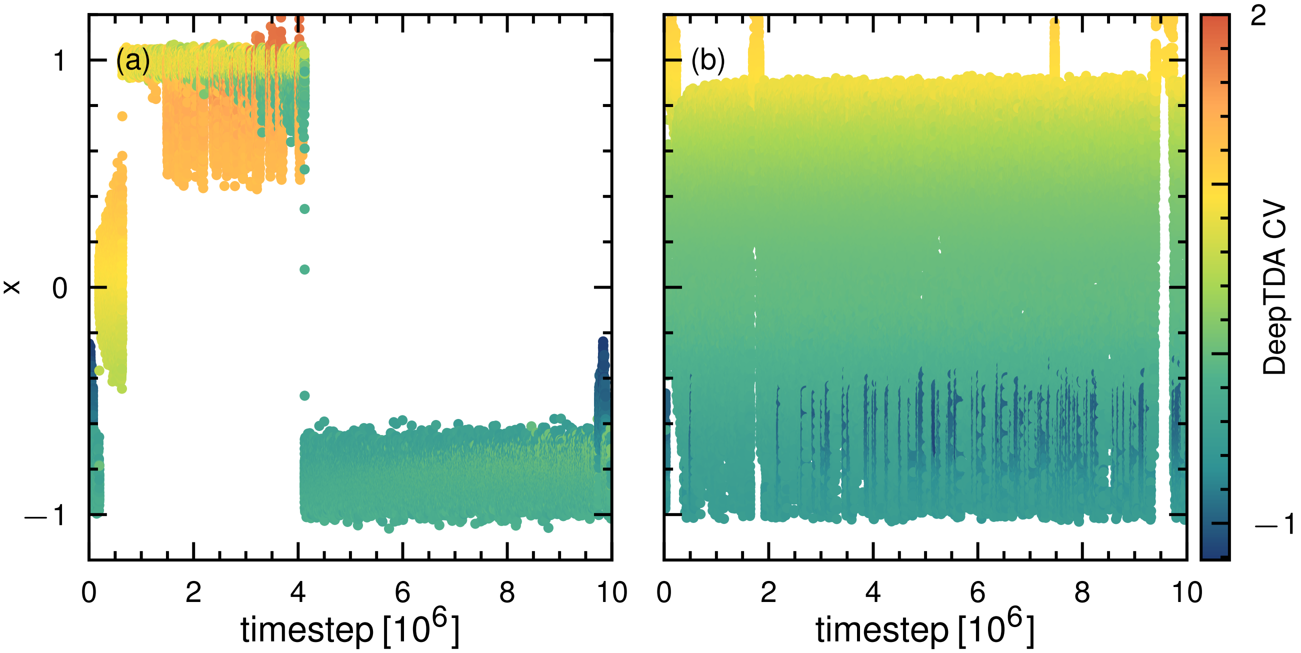

Fig. S7. Time evolution of the x coordinate of the 2D model potential, during biased simulations in configurational space using the (a) 2-state and (b) 4-state DeepTDA CVs, colored according to respective CV value.

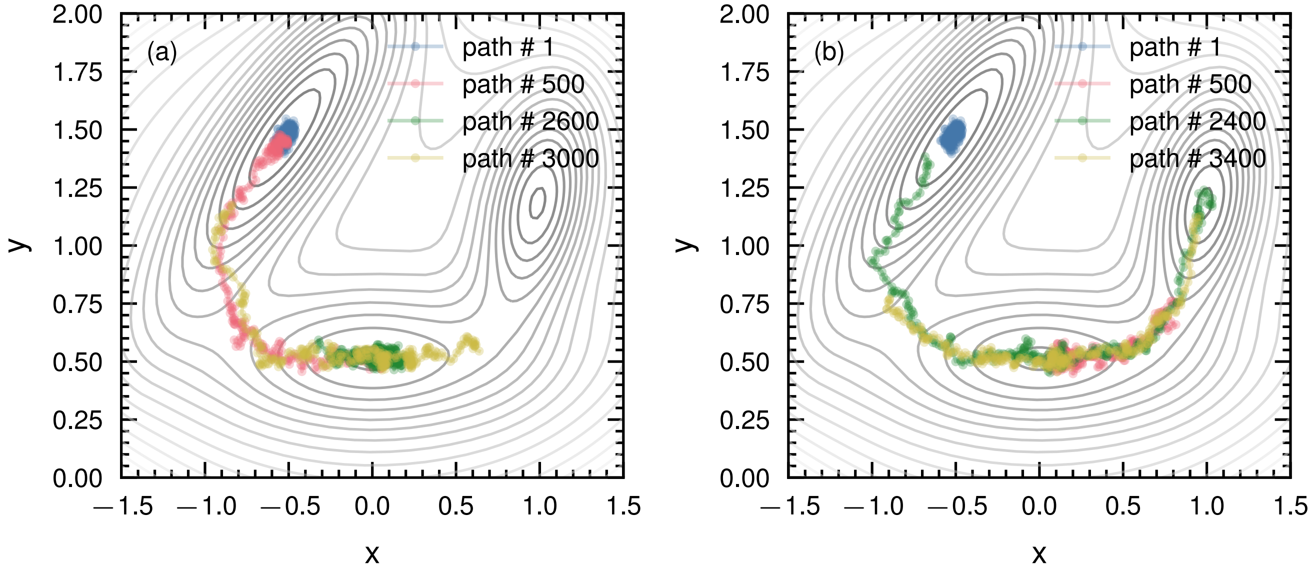

Fig. S8. Polymer snapshots of the MoP simulations using (a) the 2-state DeepTDA CV and (b) the 4-state DeepTDA CV on the 2D model potential. The equilibrated initial trajectory is shown in blue.

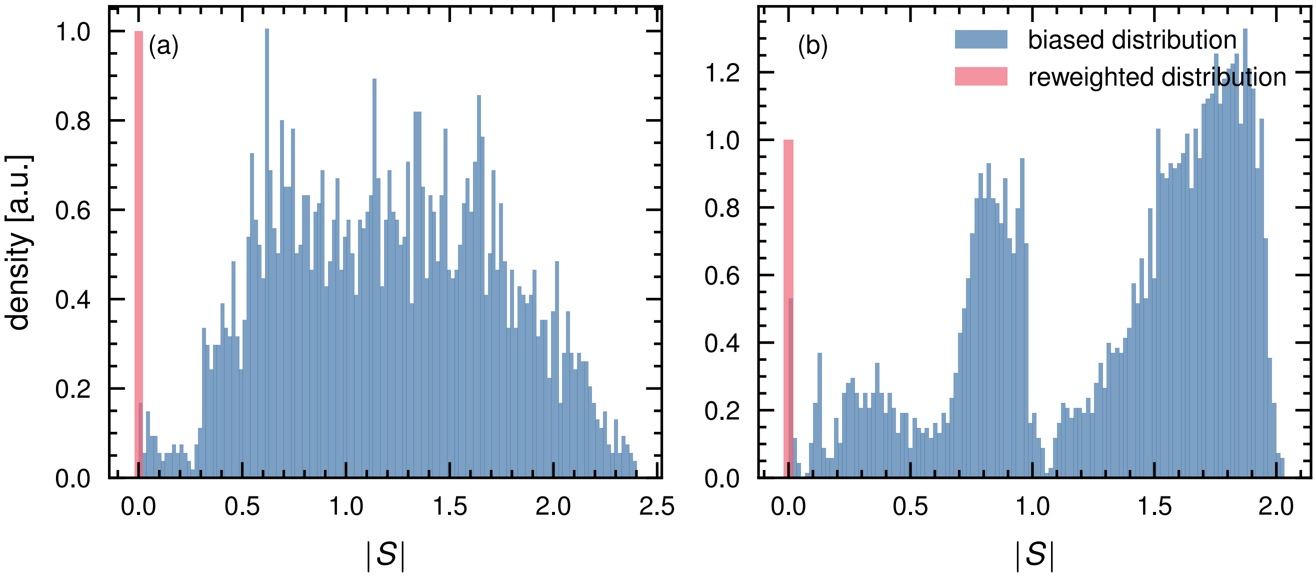

Fig. S9. The biased distribution of $|S|$ values in all polymers sampled by MoP using (a) the 2-state and (b) the 4-state DeepTDA CV on the 2D model potential is shown in blue. In panel (b), the structure centered at $|S| \approx 0.8$ corresponds to partial reactive paths connecting basin A to B or B to C of the 2D model potential, while the structure centered at $|S| \approx 1.9$ corresponds to complete reactive paths connecting A to C. In red, we report the distribution obtained after reweighting following the scheme of Ref. (1), showing the expected delta-like peak around $|S| \approx 0$. The reweighted distribution has been scaled for clarity of presentation. We note that in this work, MoP was used in an exploratory manner with the sole purpose of generating reactive trajectories. Therefore, these distributions are not converged.

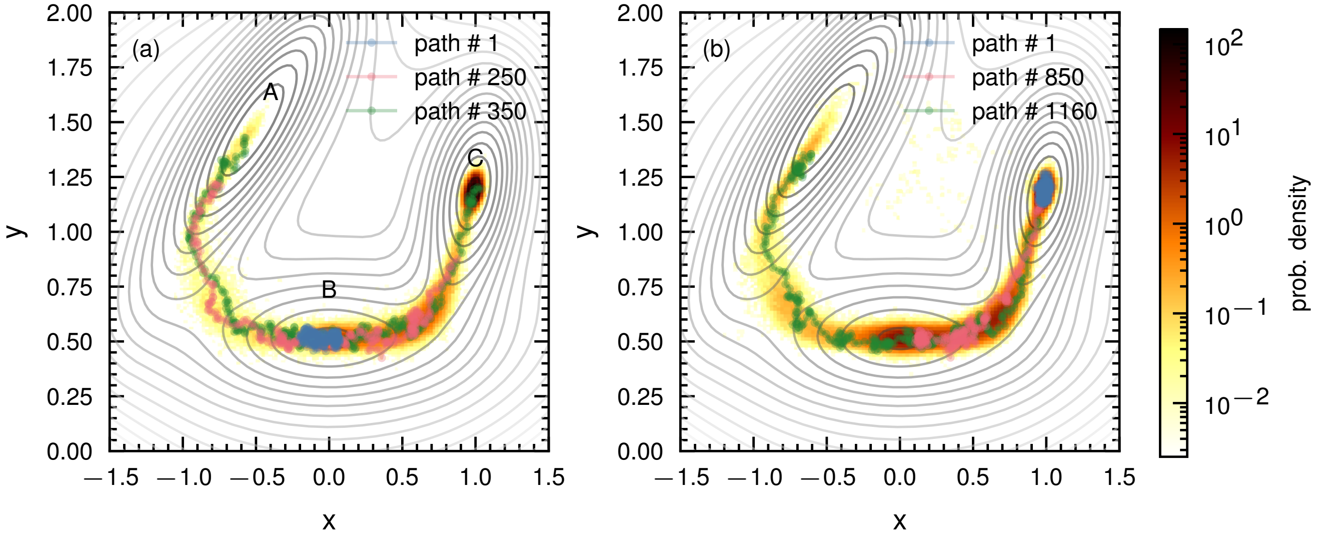

Fig. S10. MoP simulations on the 2D model potential starting from (a) basin B and (b) basin C, shown as normalized densities of all configurations in the polymers in logarithmic scale. Snapshots of selected trajectories are also shown, including the initial equilibrated trajectory (blue), and a complete reactive trajectory (green).

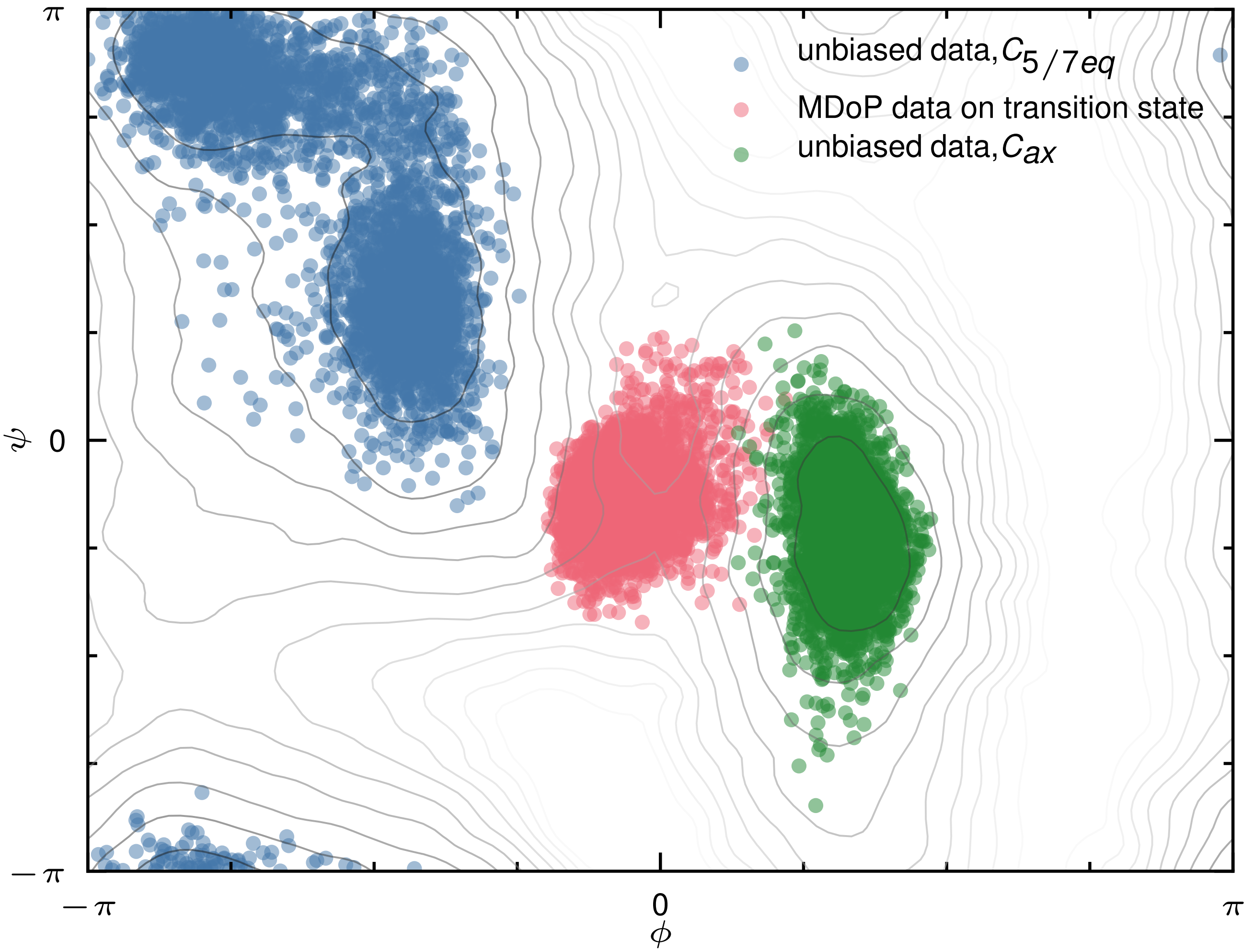

Fig. S11. Training data for the 3-state DeepTDA CV in the case of alanine dipeptide. Scatter plot of the training data, with data from unbiased simulations in the $C_{5,7eq}$ and C_{ax} basins shown respectively in blue and green, and selected configurations from reactive paths generated by MoP shown in pink. From all 3 states, 4000 configurations are shown and used to train the 3-state DeepTDA CV. Isolines of the FES calculated from a converged reference calculation using the ϕ, ψ dihedral angles are indicated in grey.

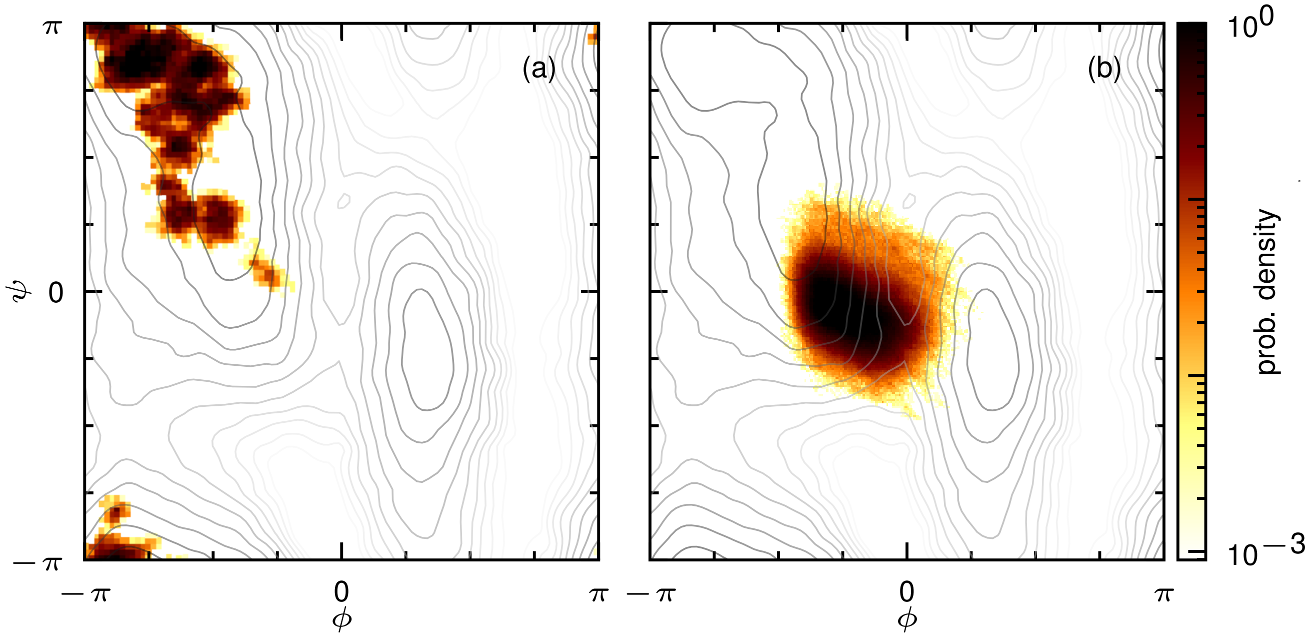

Fig. S12. Trapped (a) and reactive (b) trajectories, isolated from a MoP simulation using the 2-state DeepTDA CV for alanine dipeptide in trajectory space. The normalized densities are calculated from 125 trapped and 3529 reactive trajectories, respectively. Isolines of the FES of alanine dipeptide calculated from a converged reference calculation using the ϕ, ψ dihedral angles are indicated in grey.

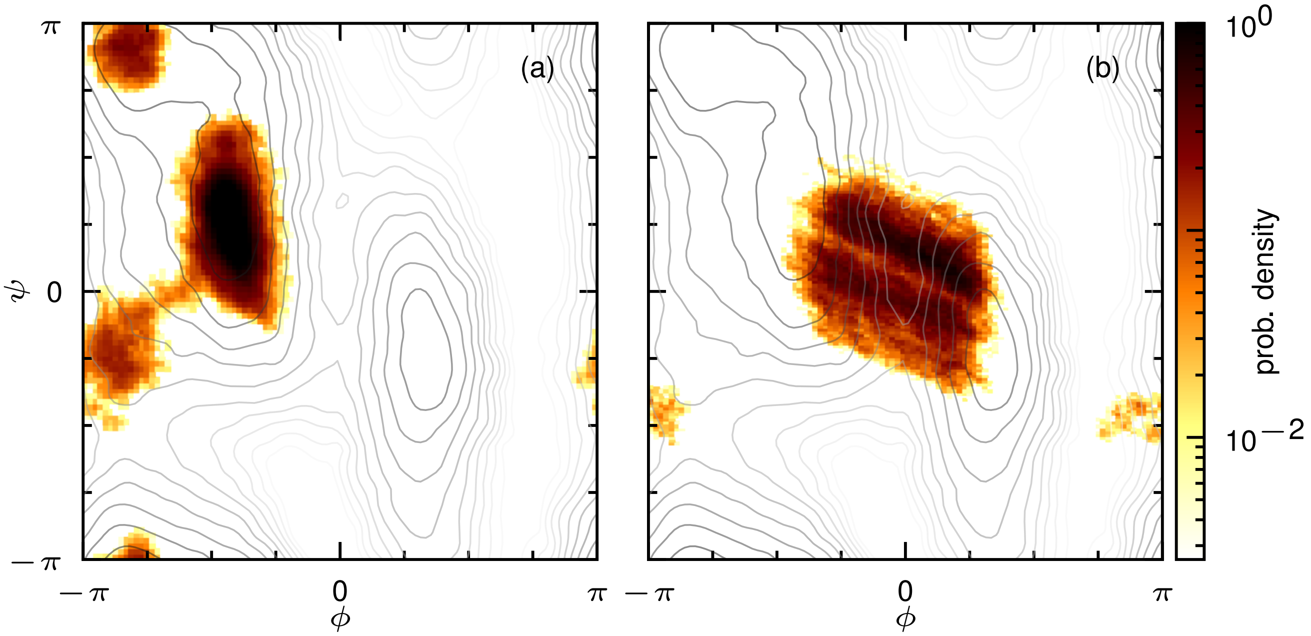

Fig. S13. Trapped (a) and reactive (b) trajectories, isolated from a MoP simulation using the 3-state DeepTDA CV for alanine dipeptide in trajectory space. The normalized densities are calculated from 1405 trapped and 569 reactive trajectories, respectively. Isolines of the FES of alanine dipeptide calculated from a converged reference calculation using the ϕ, ψ dihedral angles are indicated in grey.

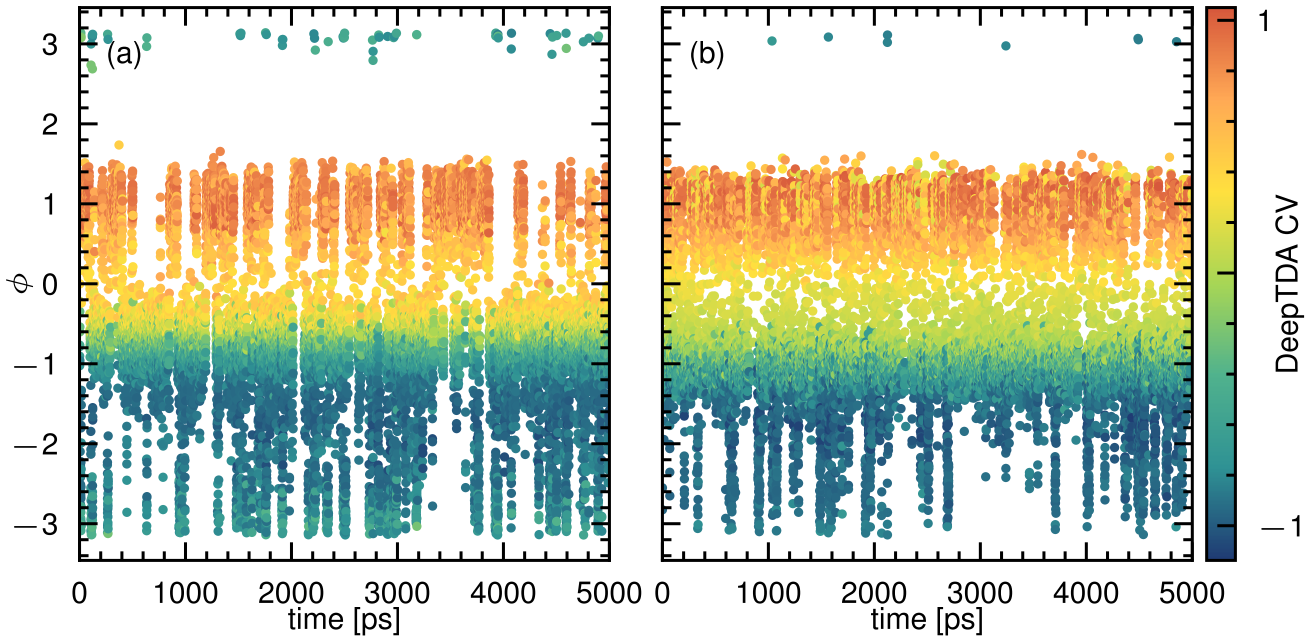

Fig. S14. Time evolution during biased simulations in configurational space using the (a) 2-state and (b) 3-state DeepTDA CVs, colored according to respective CV value.

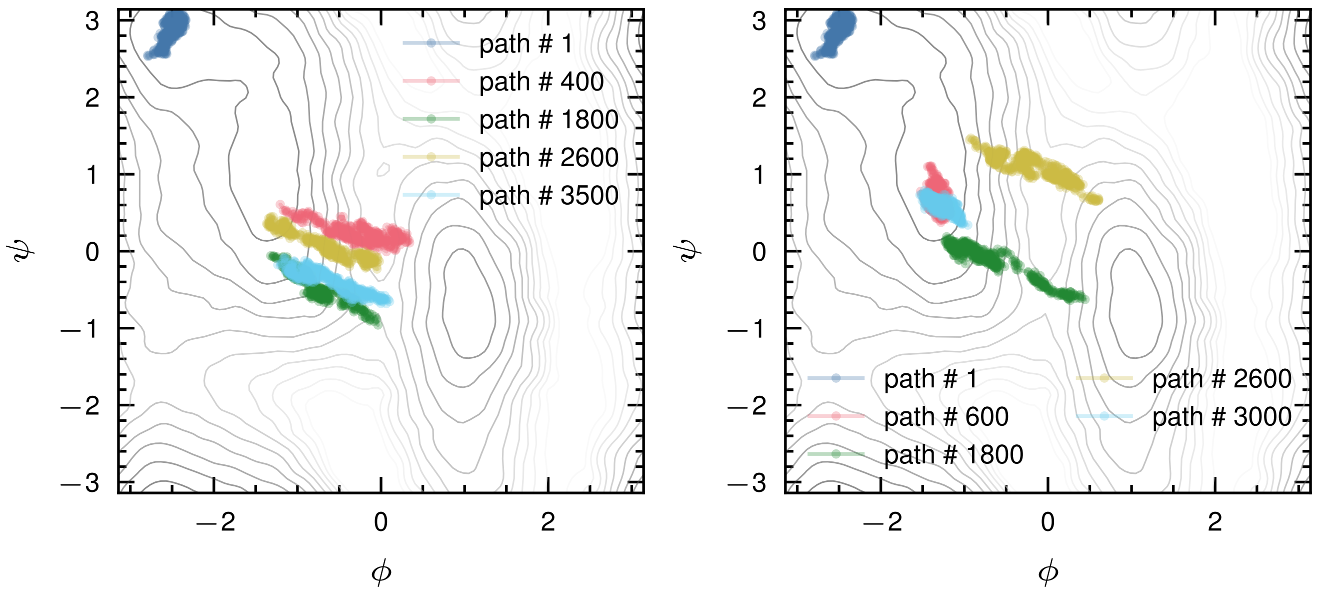

Fig. S15. Polymer snapshots of the MoP simulations using (a) the 2-state DeepTDA CV and (b) the 3-state DeepTDA CV on alanine dipeptide. The equilibrated initial trajectory is shown in blue.

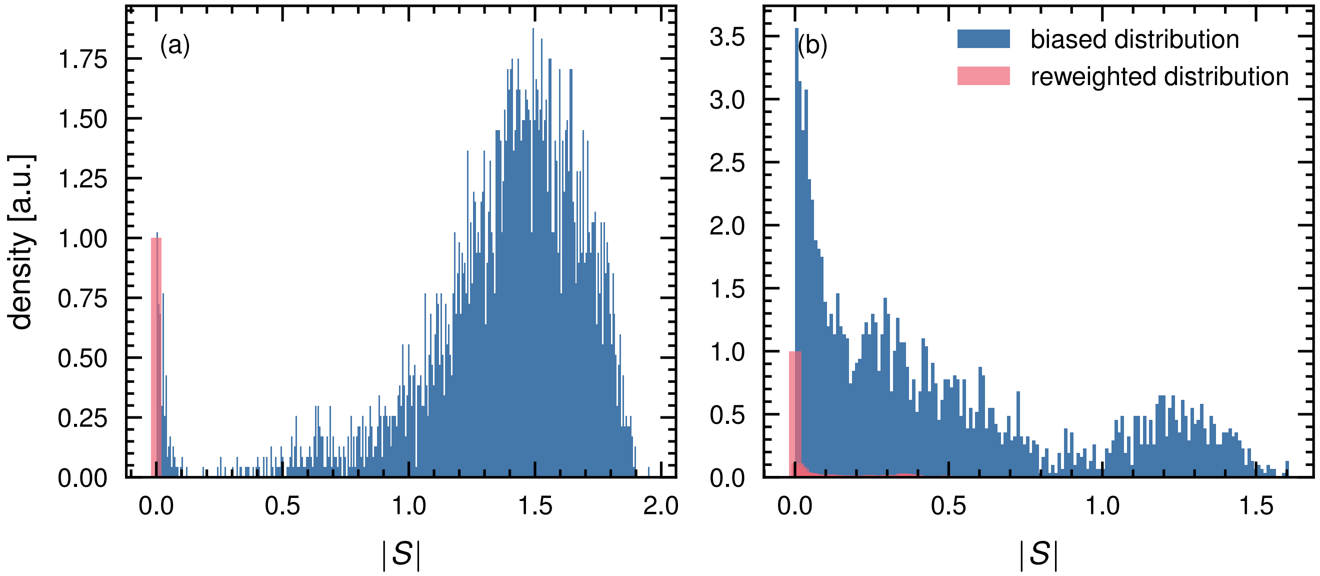

Fig. S16. The biased distribution of $|S|$ values in all polymers sampled by MoP using (a) the 2-state and (b) the 3-state DeepTDA CV on alanine dipeptide is shown in blue. In red, we report the distribution obtained after reweighting following the scheme of Ref. (1), showing the expected delta-like peak around $|S| \approx 0$. The reweighted distribution has been scaled for clarity of presentation. We note that in this work, MoP was used in an exploratory manner with the sole purpose of generating reactive trajectories. Therefore, these distributions are not converged.

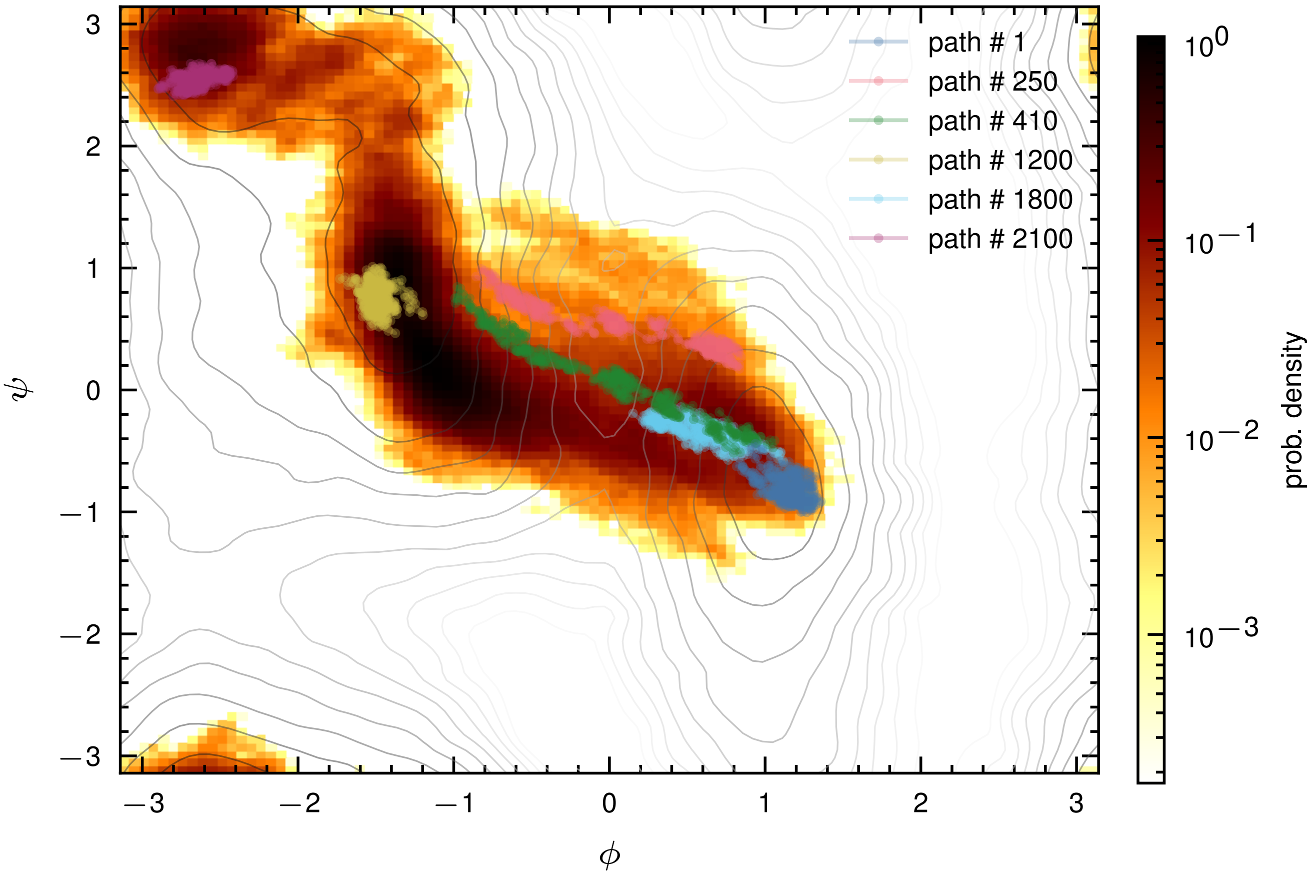

Fig. S17. MoP simulations on Alanine Dipeptide, starting from the Cax basin, shown as normalized densities of all configurations in the polymers in logarithmic scale. Snapshots of selected trajectories are also shown, including the initial equilibrated trajectory (blue), and a complete reactive trajectory (green).

References

1. M Invernizzi, M Parrinello, Rethinking Metadynamics: From Bias Potentials to Probability Distributions. *The J. Phys. Chem. Lett.* **11**, 2731–2736 (2020).
