# Supplementary figures and images for: Effective data-driven collective variables for free energy calculations from metadynamics of paths
Source: PNAS Nexus. 2024 Apr 12;3(4):pgae159. doi: 10.1093/pnasnexus/pgae159 (PMC11044970; doi:10.1093/pnasnexus/pgae159)

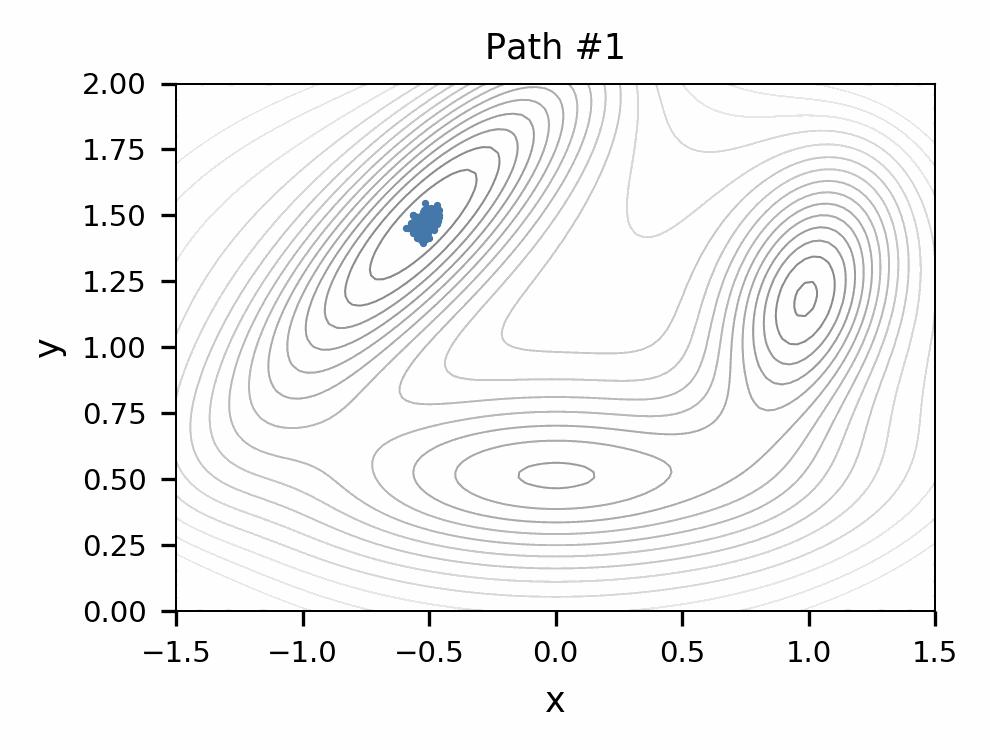

Supplement: pgae159_Supplementary_Data [file pgae159_supplementary_data.zip › video_M1.gif]

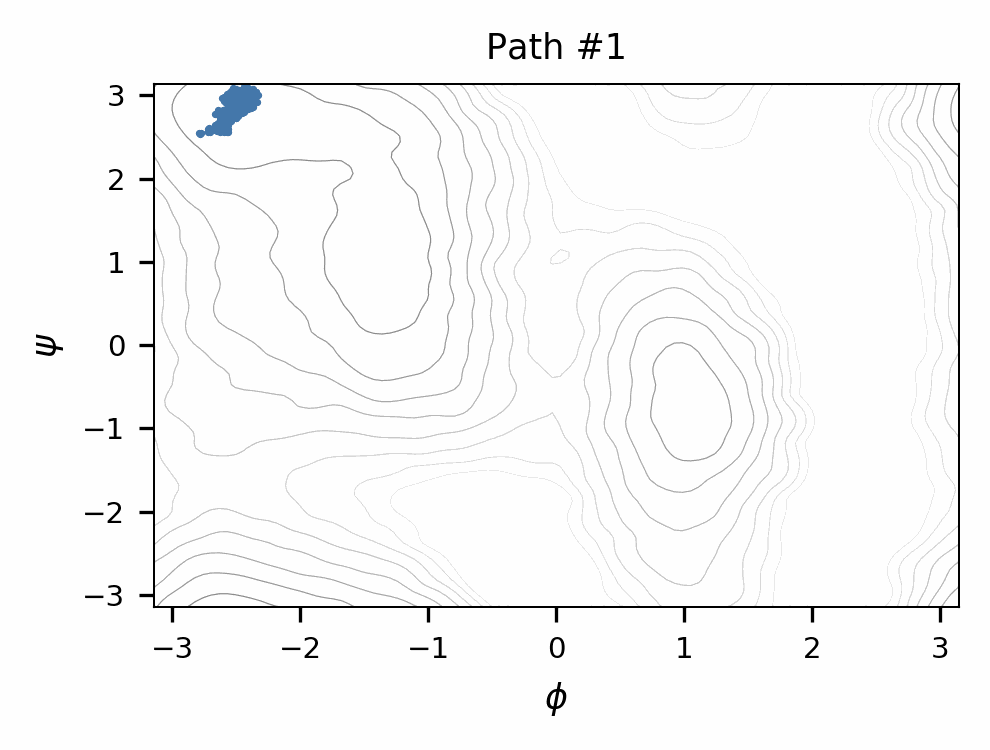

Supplement: pgae159_Supplementary_Data [file pgae159_supplementary_data.zip › video_M2.gif]
